# Comparison of first dimension IPG and NEPHGE techniques in two-dimensional gel electrophoresis experiment with cytosolic unfolded protein response in Saccharomyces cerevisiae

**DOI:** 10.1186/1477-5956-11-36

**Published:** 2013-07-27

**Authors:** Rimantas Slibinskas, Raimundas Ražanskas, Rūta Zinkevičiūtė, Evaldas Čiplys

**Affiliations:** 1 Department of Eukaryote Gene Engineering, Institute of Biotechnology, Vilnius University, V. Graiciuno 8, Vilnius LT-02241, Lithuania

## Abstract

**Background:**

Two-dimensional gel electrophoresis (2DE) is one of the most popular methods in proteomics. Currently, most 2DE experiments are performed using immobilized pH gradient (IPG) in the first dimension; however, some laboratories still use carrier ampholytes-based isoelectric focusing technique. The aim of this study was to directly compare IPG-based and non-equilibrium pH gradient electrophoresis (NEPHGE)-based 2DE techniques by using the same samples and identical second dimension procedures. We have used commercially available Invitrogen ZOOM IPGRunner and WITAvision systems for IPG and NEPHGE, respectively. The effectiveness of IPG-based and NEPHGE-based 2DE methods was compared by analysing differential protein expression during cytosolic unfolded protein response (UPR-Cyto) in *Saccharomyces cerevisiae*.

**Results:**

Protein loss during 2DE procedure was higher in IPG-based method, especially for basic (pI > 7) proteins. Overall reproducibility of spots was slightly better in NEPHGE-based method; however, there was a marked difference when evaluating basic and acidic protein spots. Using Coomassie staining, about half of detected basic protein spots were not reproducible by IPG-based 2DE, whereas NEPHGE-based method showed excellent reproducibility in the basic gel zone. The reproducibility of acidic proteins was similar in both methods. Absolute and relative volume variability of separate protein spots was comparable in both 2DE techniques. Regarding proteomic analysis of UPR-Cyto, the results exemplified parameters of general comparison of the methods. New highly basic protein Sis1p, overexpressed during UPR-Cyto stress, was identified by NEPHGE-based 2DE method, whereas IPG-based method showed unreliable results in the basic pI range and did not provide any new information on basic UPR-Cyto proteins. In the acidic range, the main UPR-Cyto proteins were detected and quantified by both methods. The drawback of NEPHGE-based 2DE method is its failure to detect some highly acidic proteins. The advantage of NEPHGE is higher protein capacity with good reproducibility and quality of spots at high protein load.

**Conclusions:**

Comparison of broad range (pH 3–10) gradient-based 2DE methods suggests that NEPHGE-based method is preferable over IPG (Invitrogen) 2DE method for the analysis of basic proteins. Nevertheless, the narrow range (pH 4–7) IPG technique is a method of choice for the analysis of acidic proteins.

## Background

Two-dimensional gel electrophoresis (2DE) is one of the most widely used technique for the global protein separation and quantification [1,2]. More than 35 years ago, 2DE was developed independently by Klose [3] and O’Farrell [4], representing the combination of two orthogonal separation techniques. In the first dimension, the proteins are separated by isoelectric focusing (IEF) according to their isoelectric point. In the second dimension, proteins are separated according to their electrophoretic mobility by conventional SDS-PAGE. There are two different first dimension separation techniques: the method of Klose [3] and O’Farrell [4], where the pH gradient is formed via carrier ampholytes (CA) (amphoteric, oligoaminooligocarbonic acids with high buffer capacity at their pI) during the focusing process and the method described by Bjellqvist and Görg [5-7] using immobilized pH gradient (IPG). The protocol of 2DE with IPGs is being constantly refined, featuring a number of significant advances and applications over the past 30 years [8]. Due to its simple handling and commercialization, IPG-based IEF is typically used for 2DE-based proteome analysis and has widespread applications. Currently, various manufacturers provide a number of different IPG strips varying in length (7–24 cm) and pH range (narrow or broad, e.g. pH 4–7 or 3–10; linear or non-linear) [9]. In contrast, CA-based IEF, being a labour-intensive technique, failed to achieve widespread application, but is still used in more specialized laboratories.

Despite a widespread application, IPG-based 2DE method still has some limitations, especially in the analysis of basic proteins [10]. Separation of basic proteins by 2DE even now is considered as a challenge, and most of gel-based proteomic studies are being performed in the acidic range. In this regard, the CA-based 2DE method still should be considered for functional proteomics experiments in a broad pH range. The first CA-based technique described by O’Farrell was efficient mostly for acidic proteins, but later O’Farrell published the CA-based 2DE method for non-equilibrium pH gradient electrophoresis (NEPHGE) concerning the separation of basic proteins [11]. For the efficient analysis of basic proteins by this 2DE method, the proteins are applied to the anodic instead of the cathodic end of the IEF gel. This technique was further improved in the laboratory of Klose, and an updated protocol of NEPHGE-based 2DE method was finally reported in 1995 [12]. The equipment necessary for performing this technique later was made commercially available from WITA GmbH as a “WITAvision” 2DE system (detailed review in [13]). Therefore, it became possible to try out various formats (IEF gel lengths from 7 to 40 cm) of NEPHGE-based 2DE method.

The aim of this study was to directly compare IPG- and NEPHGE-based 2DE techniques by using the same samples and identical 2nd dimension procedures. For IPG-based 2DE we have chosen Invitrogen “ZOOM IPGRunner” system. This mini-gel 2DE system is simple, unexpensive and both IEF gel length (7 cm) and recommended sample buffer composition is compatible with that of NEPHGE-based 2DE “WITAvision” system. It should be noted that our results represent only usage of Invitrogen IPG-based 2DE system. It was reported that commercially available IPG strips can vary considerably, leading to marked differences in subsequent protein resolution during 2DE [14].

Earlier comparisons of IPG-based versus NEPHGE-based 2DE techniques [15,16] were made as proteome analysis experiments. Here we performed a differential expression proteomics experiment using both methods and broad (pH 3–10) gradient range on UPR-Cyto stress in yeast *Saccharomyces cerevisiae* cells. The results were compared to our previous study of the same phenomenon using Invitrogen narrow range pH 4–7 IPG strips [17]. Our data suggest that NEPHGE-based 2DE method is a method of choice for the analysis of basic proteins. The most dramatic demonstration of this statement was identification of differentially expressed highly basic protein Sis1p by NEPHGE, but not by IPG technique. However, in the acidic pH range both techniques appeared to be similar with some specific advantages and drawbacks. We hope that this study will help others to choose the most efficient system or strategy to perform their proteomics experiments.

## Results and discussion

### Overview of the protocols

The same samples of whole cell lysates from yeast cells expressing measles virus hemagglutinin (MeH) or nucleocapsid protein (MeN) and from the control yeast cells (transformed with empty vector pFGG3) were focused in a broad range (pH3-10) IPG strips (Invitrogen) and non-equilibrium pH gradient gels made according to manufacturers’ (WITA) recommendations. After equilibration, the strips and gels were applied onto uniform SDS-polyacrylamide mini-gels and run under the same conditions in “Biometra” system. The second dimension SDS-PAGE with following gel staining, scanning and image analysis steps for IPG and NEPHGE samples were performed in parallel. Therefore, the only difference between IPG- and NEPHGE-based two-dimensional electrophoresis (2DE) was the first dimension isoelectring focusing step and some deviations in equilibration protocol (IPG strips were equilibrated after, whereas NEPHGE gels before the freezing in -70°C). It allowed direct comparison of the first dimension IPG and NEPGHE techniques as other parameters, conditions and samples in both 2DE experiments were exactly the same.

**Figure 1:**
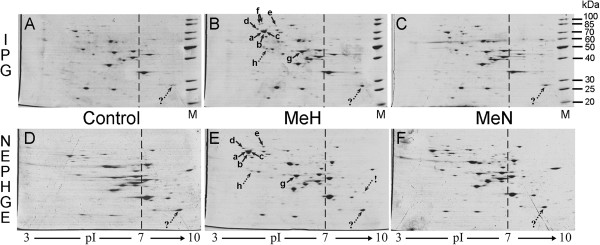
**2DE of yeast whole cell lysates using IPG (A-C) and NEPHGE (D-F) based methods at standard protein load.** The same samples from control cells **(**transformed with empty vector pFGG3; **A**, **D)** and MeH **(**pFGG3-MeH transformant; **B**, **E)** or MeN **(**pFGG3-MeN; **C**, **F)** expressing cells were loaded onto IPG strips (50 μg of total protein in each strip) and NEPHGE gels (30 μg of total protein in each gel). Approximate pI values are indicated below the gels (pH 3–10 gradient was used in both methods). Dashed line indicates approximate zone of neutral pI 7.0, which separates acidic (on the left, pI < 7) and basic (on the right, pI > 7) protein spots. Protein molecular weight markers (M) are loaded onto IPG-based 2D gels, their masses are indicated at the right (kDa). Arrows point to the spots described in Table 3. Solid arrows indicate protein spots that were identified in our previous work [17], whereas dotted arrows point to additional spots identified by MS in this study. Quantitative analysis of each indicated protein spot is presented in Table 3.

**Figure 2:**
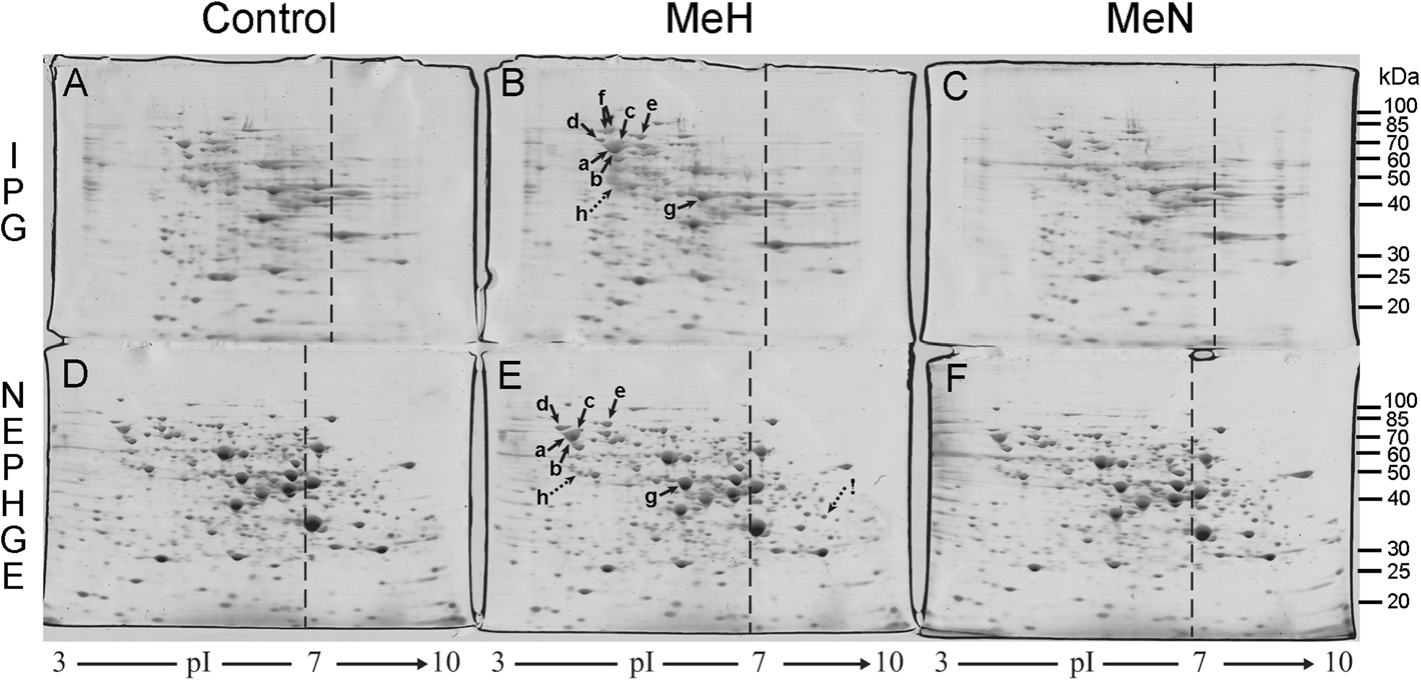
**2DE of yeast whole cell lysates using IPG (A-C) and NEPHGE (D-F) based methods at high protein load.** The same concentrated samples from control cells **(**transformed with empty vector pFGG3; **A**, **D)** and MeH **(**pFGG3-MeH transformant; **B**, **E)** or MeN **(**pFGG3-MeN; **C**, **F)** expressing cells were loaded onto IPG strips (100 μg of total protein in each strip) and NEPHGE gels (100 μg of total protein in each gel). Original scan of one of the replicas is shown for comparison (six gels were being scanned in parallel at the same time). The references are the same as in Figure 1.

Examples of 2D gel images are shown in Figures 1 and 2. We have analysed various protein spot parameters at two different experimental conditions: at standard 1x protein load (50 μg of whole cell lysate protein per gel, as recommended by manufacturer of IPG strips) and at high 2x protein load (100 μg of total protein per gel). General quantitative analysis of IPG- and NEPHGE-based 2DE methods is presented in Tables 1 and 2, respectively, whereas their “trial” comparison with the concrete biological experiment [17] is summarized in Table 3.

**Table 1:** Comparison of spot parameters in IPG- and NEPHGE-based 2DE at standard protein load

**Method**^**1**^	**IPG**^**2**^			**NEPHGE**^**3**^		
**Parameter**^**4**^	**pI 3-10**	**pI <7**	**pI >7**	**pI 3-10**	**pI <7**	**pI >7**
Number of detected protein spots^5^	102	79	23	110	80	30
Reproducibility of spots^6^%	75 ± 4%	82 ± 1%	44 ± 18%	76 ± 17%	72 ± 18%	87 ± 20%
Total^7^ protein volume in a gel (Vol)	410302 ± 76913	325075 ± 73300	85227 ± 13613	444930 ± 75631	278277 ± 69205	166653 ± 15835
	(100 ± 19%)	(79 ± 4%)	(21 ± 4%)	(100 ± 17%)	(63 ± 6%)	(37 ± 6%)
Variation of spot volume (ΔVol)^8^	35% ± 25	36% ± 25	30% ± 25	40% ± 33	46% ± 36	27% ± 21
Variation in relative volumes of spots (Δ%Vol)^9^	30% ± 23	30% ± 23	26% ± 19	31% ± 28	31% ± 30	31% ± 25
Average saliency of protein spot^10^	2794 ± 293	2927 ± 307	2248 ± 947	2610 ± 549	2202 ± 558	3523 ± 810
Low quality spots (saliency <500)^11^%	15 ± 3%	14 ± 6%	21 ± 11%	27 ± 8%	30 ± 9%	20 ± 9%

^1^The same samples were analysed in IPG- and NEPHGE-based 2DE systems; ~50 μg of whole cell protein was loaded onto IPG strips and ~30 μg onto NEPHGE gels (due to small space for sample application in NEPHGE tubes – see text).

^2^Immobilized pH gradient (IPG) based 2DE method (Invitrogen pH3-10 system).

^3^ Non-equilibrium pH gradient gel electrophoresis (NEPHGE) based 2DE method (WITAvision pH3-10 system).

^4^Parameters were calculated from 2–3 replicas (repeating analysis of the same samples). Each parameter was calculated both for whole gel (pI 3–10, all detected proteins) and for its pI <7 and pI >7 parts (acidic and basic proteins, respectively). Neutral pI 7, separating acidic and basic protein spots, is indicated by dashed line in Figure 1.

^5^Number of detected separate protein spots in all samples (Control, MeH ir MeN), from all replicas.

^6^The same spots detected among replicas of the same sample (according to matches of the spots generated by 2D image analysis software *ImageMaster 2D Platinum 7.0*); the percentage of matched spots (±SD) is given for a whole gel (pI 3–10) and for its acidic or basic parts.

^7^Total volume (*Vol*; product of spot area and intensity) of all protein spots in one gel is calculated by 2D analysis software; here the average from whole pI3-10 gel is given as 100% (±SD), whereas pI <7 and >7 indicate acidic and basic protein portions, respectively.

^8^Variation of volumes of the same spots in separate replicas; calculation was made using all spots matched by the software and then the average of variation ΔVol ±SD was calculated.

^9^%*Vol* indicates percentage of volumes of separate spots among volume of all protein spots in a gel. In this case, all matched protein spots were evaluated in the same way as calculating variation of volumes (8), only instead *Vol* the values of %*Vol* was used (the result is average of Δ%Vol ± SD).

^10^Average saliency of detected protein spots per gel ± SD.

^11^Detected protein spots with saliency <500 were considered as low quality spots (see text). The percentage of such protein spots (±SD) was calculated for a whole gel (pI 3–10) and for its acidic or basic parts.

**Table 2:** Comparison of spot parameters in IPG- and NEPHGE-based 2DE at high protein load

**Method**^**1**^	**IPG**^**2**^			**NEPHGE**^**3**^		
**Parameter**^**4**^	**pI 3-10**	**pI <7**	**pI >7**	**pI 3-10**	**pI <7**	**pI >7**
Number of detected protein spots^5^	432	321	111	506	372	134
Reproducibility of spots^6^%	68 ± 1%	73 ± 4%	51 ± 13%	87 ± 5%	85 ± 6%	90 ± 4%
Total^7^ protein volume in a gel (Vol)	1726878 ± 260176	1357575 ± 226314	369303 ± 59726	2618475 ± 58090	1845417 ± 54127	773057 ± 30071
	(100 ± 15%)	(79 ± 3%)	(21 ± 3%)	(100 ± 2%)	(70 ± 1%)	(30 ± 1%)
Variation of spot volume (ΔVol)^8^	49% ± 55	48% ± 54	55% ± 58	26% ± 31	28% ± 34	22% ± 23
Variation in relative volumes of spots (Δ%Vol)^9^	47% ± 51	46% ± 50	53% ± 57	25% ± 31	27% ± 34	21% ± 22
Average saliency of protein spot^10^	1931 ± 348	2028 ± 366	1419 ± 348	3210 ± 136	2947 ± 307	4189 ± 205
Low quality spots (saliency <500)^11^%	20±5%	18±5%	28±7%	11±4%	13±5%	6±3%

2x higher protein amounts were loaded onto 1st dimension gels, than for standard application described in Table 1. Preparation of concentrated whole cell lysates for this experiment is described in Methods section. Other procedures and all calculations were the same as for 1x protein load described in Table 1. An example of 2D gel images from a high load experiment is shown in Figure 2.

^1^The same samples were analysed in IPG- and NEPHGE-based 2DE systems; the equal amounts of ~100 μg of whole cell protein were loaded onto IPG strips and onto NEPHGE gels.

^2–11^The references are the same as in Table 1 and all parameters were calculated exactly as described in Table 1 legend.

**Table 3:** **Quantitative analysis of differentially expressed protein spots by 2DE using pH3-10 range (this study) and pH4-7 platform (previous work, ref.**[17]**)**

**No.**^**1**^	**Ref.**^**2**^	**Name**^**3**^	**IPG 4-7**^**4**^	**IPG 3-10**^**5**^		**Nephge 3-10**^**6**^	
				**Standard**^**5**^	**High load**^**5**^	**Standard**^**6**^	**High load**^**6**^
a	1	SSA1/2	2.4 ± 0.2	1,6 ± 0,1	1,6 ± 0,4	2,6 ± 0,3	2,0 ± 0,2
b	2	SSA1/2					
c	3	SSA4					
d	4	KAR2	3.8 ± 0.4	2,7 ± 0,5	1,8 ± 0,4	9,0 ± 3,1	2,5 ± 0,2
e	5	SSE1	2.3 ± 0.2	1,4 ± 0,3	1,8 ± 0,7	2,2 ± 0,8	1,7 ± 0,1
f	6	HSC82	2.1 ± 0.3	2,0 ± 0,2	2,1 ± 1,0	- -	- -
	6	HSP82					
g	7	ENO2	1.5 ± 0.2	1,4 ± 0,3	1,1 ± 0,2	1,3 ± 0,2	1,1 ± 0,1
h	N.I.^7^	SSA1/2^7^	2.2 ± 0.3	1,5 ± 0,1	1,1 ± 0,1	1,6 ± 0,3	2,1 ± 0,4
?^8^	N.A.	GPM1	N.A.	2,2 ± 1,3	0,7 ± 0,2	1,3 ± 0,3	1,0 ± 0,1
!^8^	N.A.	SIS1	N.A.	- -	- -	2,6 ± 0,4	2,2 ± 0,2

^1^Differentially expressed protein spots in this experiment are indicated by letters (see Figures 1 and 2).

^2^The same protein spots are indicated by the numbers in the referenced article ([17], see Figure nine and Table one).

^3^Accepted name from the *Saccharomyces* genome database (SGD) and YPD. Spots 1 and 2 represent mixtures of similar proteins Ssa1 and Ssa2 (97% identity) at an unknown ratio (see Table one legend in [17]).

^4^Cellular protein expression fold change in MeH expressing versus control cells that was determined in previous work using pH4-7 IPG-based 2DE system (Invitrogen); the values are taken from the Table one in reference [17].

^5^Expression fold changes of the same proteins determined from independent experiments in this work using pH3-10 IPG strips (Invitrogen); ~50 μg of whole cell protein was loaded onto IPG strips in “Standard” experiment, whereas ~100 μg was used in a “High load” experiment.

^6^Expression fold changes of the same proteins determined from independent experiments in this work using pH3-10 NEPHGE first dimension gels (WITAvision); ~30 μg of whole cell protein was loaded onto NEPHGE gels in “Standard” experiment, whereas ~100 μg was used for each gel in the “High load” analysis.

^7^Not identified (N.I.) in previous study, because increased amount of this protein was observed only in cells expressing MeH, but not MuHN protein (the expression fold change in MeH/Control cells determined by IPG4-7 system here is given from our unpublished data).

^8^? and ! indicate basic protein spots (pI > 7) that were not analysed in previous experiment on pH4-7 platform (N.A. – not assayed). Despite that protein spot “?” showed false expression change (“artefact”) in IPG-based system (unreliable expression changes are apparent by high error range), in this experiment it was identified as phosphoglycerate mutase 1 (Gpm1p). Protein Sis1p in this study was identified using NEPHGE-based 2DE system, whereas it was not detected by IPG-based 2DE method.

### Handling differences

When comparing IPG- and NEPHGE-based methods, the specific differences between these procedures should be mentioned. In the case of IPG, we used commercial dried polyacrylamide gels with immobilized pH gradient attached to plastic strips. After application of the sample, gel is left to rehydrate overnight, and during this step proteins enter the gel. Following rehydration, the first dimension electrophoresis – isoelectric focusing (IEF) – is performed in IPG strips, and proteins usually reach their isoelectric point, where their charge equals to zero. Such IEF procedure could be defined as equilibrium pH gradient electrophoresis. The protocol for this method is simple and easy, because IPG strips are practically identical, and convenient, well-defined procedure is used in every experiment. Therefore, it is easy to repeat the procedure in exactly the same way, and repeatable results can be expected. In the case of NEPHGE, the first dimension gels are casted by the user himself and the gel length and quality (e.g. presence or absence of the bubbles in the gel, etc.) depends only on the handiness of the experimenter. Moreover, after 1st dimension IEF, the handling of IPG strips is safe and easy, whereas NEPHGE tubal gels are fragile, could be easily broken during extrusion from the tubes, slipped out from equilibration grooves and are fragmented into pieces under careless treatment. Therefore, in terms of simplicity the IPG method is preferable over NEPHGE, which could be called “stressful” and requires serious skills. However, despite some troubles with NEPHGE gels in our experiments (in the beginning we have been loosing about half of the 1st dimension gels), the results presented here demonstrate the applicability of this method.

### Spot reproducibility

We used Coomassie staining of 2D gels. It is not very sensitive protein detection method, and in this case manufacturer of IPG strips (Invitrogen) recommends loading 20–50 μg of total protein per ZOOM strip. For “standard” loading experiment, we used maximal recommended protein amount (50 μg) per strip. It should be mentioned that smaller amount of the whole cell protein was used in parallel NEPHGE-based 2DE experiment (~30 μg versus ~50 μg in IPG). The volume of the sample is limited by the narrow tube diameter in NEPHGE procedure; therefore, it is impossible to load more protein if sample is too diluted. An example of 2D gels from this experiment is shown in Figure 1. Under these conditions, IPG- and NEPHGE-based 2DE methods were compared quantitatively in the Table 1. Similar number of protein spots (~100) was detected in 2D gels using both methods. As the study using standard protein load did not represent enough yeast proteins to make firm conclusions, we repeated the experiment by loading double amounts of protein on each gel. For this experiment, we used more concentrated whole cell lysates (see Methods). The same experimental variants were analysed by loading 100 μg of whole cell protein onto each IPG strip and NEPHGE gel. All other 2DE conditions were exactly the same. The number of detected spots at high protein load substantially increased. More than 400 different spots were detected by IPG- and over 500 spots by the NEPHGE-based 2DE (an example of 2D gels is shown in Figure 2, whereas quantitative analysis at high protein load is presented in Table 2).

A comparison of loaded and detected protein amounts suggests that more than 1/3 of total protein amount was lost using IPG strips in comparison to the total volume of protein spots detected in NEPHGE-based 2D gels (Tables 1 and 2). Analysis of the separate parts of 2D gels reveals that the loss of total protein in IPG-based 2DE method is mostly determined by the loss of basic proteins. In Tables 1 and 2 it is shown that protein amount in 2D gels is distributed unequally: in the case of NEPHGE, detected basic protein amount is twice as large as in IPG-based 2D gels, whereas total volume of acidic protein spots is somewhat similar in both techniques.

The drawbacks of IPG method in the basic gel side are not limited to the protein amount. Different proteins are lost in separate experiments; it is obviously demonstrated by the reproducibility parameter in Tables 1 and 2. Only about a half of basic protein spots detected by IPG-based method (~44% and ~51% at standard and high protein load, respectively) are reproduced, still with large variation. Therefore, in IPG-based 2DE experiment, it is possible to quantitatively evaluate only ~50% of detected basic protein spots, and even these tend to show unreliable results (described below). In contrast, the reproducibility of NEPHGE-based method is best in the basic gel side with the reproducibility of ~90% and minimal gel-to-gel variation at high protein load (Table 2). A few spots in NEPHGE-based 2D gels at standard protein load were not evaluated due to our imperfect performance in the first dimension with a couple of the control sample replicas. Some bubbles introduced during loading of the sample or slightly shorter 1st dimension NEPHGE gel resulted in incomplete focusing or impaired spot resolution (Figure 1, D or not shown). These problems were avoided when running NEPHGE samples at high protein load.

The analysis of acidic proteins shows good reproducibility in the IPG-based method at standard protein load, because >80% spot reproducibility practically coincides with variations of total protein amount in the gel, which in our case reached almost 20% (due to loading errors or differences in gel staining intensities). Lesser amount of protein on the gel results in dissapearance of weak spots, and this is the main reason of the differences among the replicas. It is evident from Figure 1 that protein pattern in 2D gels of different samples (Control, MeH, MeN) analysed by the same method is very similar. By comparing our 2D gel images obtained using IPG-based method with earlier IPG-2DE analyses of yeast proteome [18,19], it could be noticed that positions and relative amounts of the vast majority of protein spots in our experiments match well with the results of previous studies. The exceptions are protein spots differently expressed due to different experimental conditions. Surprisingly, high protein load experiment showed better reproducibility of acidic protein spots in NEPHGE- than in IPG-based 2DE, and the difference was significant. This resulted in considerable difference of overall spot reproducibility with ~87% in NEPHGE- versus only ~68% in IPG-based 2DE (Table 2). It suggests that IPG strips were overloaded. Indeed, some areas in acidic gel zone showed incomplete focusing and loss of resolution in IPG-based 2D gels at high protein load (see Figure 2, upper panel).

### Spot quality and protein capacity of the 1st dimension gels

Spot quality is also an important parameter in 2DE analysis. We used ImageMaster 2D Platinum 7.0 software (GE Healthcare), which calculates saliency value for every detected protein spot. This parameter is a measure based on the spot curvature. Real spots generally have high saliency values, whereas artifacts and background noise have small saliencies. The saliency is an efficient parameter for filtering and discarding spots, but it may also be used for the evaluation of the spot quality. Other 2D gel analysis software packages provide some “spot quality” values, which are also based on the spot curvature property. For example, PDQuest software (BioRad) calculates spot quality numbers, which are mainly based on Gaussian fit assessing spot shape. Absolute values of spot saliency may vary depending on brightness and contrast of 2D images. However, in our case all procedures of image processing for both IPG- and NEPHGE-based 2DE gels were the same, and therefore it was possible to compare two methods by using saliency as the spot quality parameter. We have calculated the average saliency of protein spot and the percentage of low quality spots for every gel. This data is provided in Tables 1 and 2. Average spot saliency for a whole gel at standard protein load was similar in both methods, but again there was a difference when comparing acidic and basic gel zones. Quality of acidic protein spots was higher in IPG, whereas basic proteins were better shaped in NEPHGE-based 2DE method (Table 1). To count low quality spots, we had set an arbitrary value of saliency to 500. The saliency is highly dependent on the images, and, according to the software user manual, gels may need saliency values from 10 to 5000 for correct filtering. We have discarded all spots with a saliency <150, whereas protein spots with a saliency of <500 were defined as low-quality spots. The percentage of low-quality spots of acidic proteins at standard 1x protein load was considerably higher in NEPHGE-based method, whereas the results for spots of basic proteins were similar in both methods (Table 1). However, high protein load experiment showed substantially different results. Spot quality data confirmed that IPG strips were overloaded by 2x total protein amount. Thus, double protein load significantly decreased average spot saliency and increased percentage of low quality spots in IPG-based 2D gels, whereas NEPHGE-based 2D gels demonstrated increased overall spot quality (see Table 2 and compare with Table 1). Especially convincing was the reduction of low quality spots in NEPHGE gels, with only ~6% of detected basic protein spots found below saliency value of 500 (Table 2). Lower spot quality in standard protein load experiment may be at least partially explained by our imperfect performance with NEPHGE gels, which is reflected by higher error ranges of average saliency values than at high protein load (Tables 1 and 2, respectively). Anyway, high protein load onto NEPHGE gels is preferable, because both spot quality and reproducibility are excellent. It seems that loading 100 μg of whole cell protein onto NEPHGE gel is near to optimal amount in a mini-gel format using Coomassie staining. Further attempts to increase protein amount and detect even more spots in a small gel may result in overlaping of neighbouring proteins by spots of high-abundance proteins.

Experiments with loading 1x and 2x protein amount per gel revealed different protein capacity of IPG strips and NEPHGE gels. Recommendations of manufacturer to load only up to 50 μg of total protein onto broad pH range IPG strip seems to be correct, because double protein amount resulted in overloading and loss of spot quality and reproducibility. Therefore, protein capacity of pH 3–10 IPG strip is limited to ~50 μg of total protein. Meanwhile, the protein capacity of NEPHGE gel is at least ~100 μg of total protein from the same sample. It should be noted that higher protein capacity of NEPHGE gels over IPG strips is not limited to ~2 fold. The volume of NEPHGE gels is much smaller than volume of IPG strips of the same length. The volume of NEPHGE tubal gel (7 cm in length and 0.9 mm in diameter) is only ~45 mm^3^, whereas IPG gel (70 mm × 0.5 mm × 3.3 mm) has a volume of ~115 mm^3^. Two and half fold difference in volumes means that protein capacity of the same volume NEPHGE gel is ~5 fold higher than that of a broad range 1st dimension IPG gel.

### Impact of the procedure on experimental variations

The results of the quantitative analysis showed considerable variation in the relative volumes (%Vol) of the spots among experimental replicas of the same samples at standard protein load, constituting ~30 ± 25% (Table 1). It means that ~1.3 ± 0.3 fold change of the %Vol of a protein spot between different experimental conditions is in the range of error and should not be estimated as biological effect. We noticed that at least half of this variation is determined by different conditions in independent experiments. Comparing the same samples processed in parallel, the variation in %Vol constituted only 10-15%. Thus, the easiest way to minimize the variation is to compare experimental sample with the control processed in parallel, but not in separate independent experiments. Experimental variation itself should not introduce false positive results when using larger amount of replicas, because in this case it is apparent in the error range. However, it should be considered that the threshold of fold change for differentially expressed protein spot is at least 1.3 if samples are run in parallel and >1.5-1.6 fold if samples are processed in separate 2DE experiments. Lower fold changes would fall into experimental variation range and are unreliable values for differential expression. These thresholds should be considered at least when analysing whole cell lysates.

High protein load did not considerably affect experimental variations of spot volume in the case of NEPHGE, except that the average %Vol variation of basic protein spots decreased from ~31% at standard load to ~21% at high load conditions (Tables 1 and 2). All other spot variation parameters were almost identical in NEPHGE-based 2DE at both standard and high protein load. Accordingly, the threshold for considering any protein spot as differentially expressed at high load should be the same as under standard conditions. Different situation was observed in the case of IPG, where high sample load increased variation of spot volumes, reaching ~50 ± 50% (Table 2). It indicates that ~1.5 ± 0.5 fold change in the %Vol of a protein spot can be the result of experimental variation. The threshold for differential expression under such conditions should be increased to ~2.0 fold, and this may substantially complicate analysis of biological variations.

### Evaluation of procedure impact on biological variations

Both 2DE methods were examined in biological experiment on cytosolic unfolded protein response (UPR-Cyto), and the results compared with our earlier study, where the same phenomenon was analysed using narrow range (pH 4–7) Invitrogen IPG-based 2DE method [17]. Here we show the analysis of analogous samples analysed in a broad range 1st dimension pH 3–10 gradient by both IPG- and NEPHGE-based 2DE methods. The main differentially expressed protein spots, already identified and evaluated in previous experiment, are indicated by solid arrows in Figures 1 and 2, and their quantitative analysis is given in Table 3.

In previously published work comparing IPG- and NEPHGE-based 2DE methods, it was difficult to predict the protein mobility in different gel systems [15]. We suggest that this may be partially related to different sample preparation and 2nd dimension electrophoresis in each method, because in our experiment overall protein pattern is rather similar, and most corresponding spots of high abundant proteins can be cross-referenced (Figure 1). The essential results of our previous study on UPR-Cyto response were repeated by both IPG and NEPHGE pH 3–10 systems; however, the quality of results is different. At standard protein load, both pH 3–10 methods were less sensitive than pH 4–7 method in the acidic pI range. The spots of low abundant proteins Sgt2p, Sti1p and Hsp104p were quantitatively evaluated and identified in earlier pH 4–7 IPG-based 2DE study [17]. In this experiment, spots of Hsp104p were near the limit of detection, preventing reliable quantitative analysis, whereas Sgt2p and Sti1p were entirely undetectable using standard protein load in pH 3–10 based method. The increased expression of more abundant pI 4–7 proteins in UPR-Cyto was also determined by IPG pH 3–10 method; however, calculated fold changes were lower than in previous study (Table 3). New differentially expressed protein spots in the acidic pI range were not detected by either pI 3–10 range method. In this study, we have identified by mass spectrometry (MS) only one additional acidic protein - spot “h” (Figure 1), but it was also detectable in earlier pI 4–7 based 2DE analysis with even higher fold change (see Table 3). The identified mixture of similar cellular chaperones Ssa1 and Ssa2 in spot “h” does not provide new proteins in UPR-Cyto, as their major forms are represented in spots a and b. The appearance of these minor isoforms of Ssa1/2p only suggests that the main overexpressed UPR-Cyto proteins undergo partial proteolysis in MeH expressing yeast cells.

Lower sensitivity of pH 3–10 versus pH 4–7 IPG in the acidic zone could be compensated by the analysis in the basic pI protein zone. However, using IPG pI 3–10 method, we have not identified any basic protein induced in UPR-Cyto stress neither at standard, nor at high protein load. Therefore, the use of IPG pH 3–10 instead of IPG pH 4–7 system is unsuitable, as its drawbacks are not compensated by any practical advantages. Comparison of a broad pH 3–10 range IPG and NEPHGE in acidic protein zone reveals positive and negative features of both methods. Some of the main UPR-Cyto proteins showed higher fold changes in NEPHGE-based method, and, in the case of Ssa and Sse1 proteins, the results of quantitative analysis are in better agreement with the earlier pH 4–7 IPG-based analysis than with the results of pH 3–10 IPG-based method (Table 3). The main drawback of NEPHGE-based method was its failure to detect acidic UPR-Cyto protein Hsc/Hsp82 (Figures 1 and 2, spot f). In the basic side of 2D gels, the results unambigously demonstrated the advantage of NEPHGE over IPG. For example, the first replicas of IPG pH 3–10 based 2D gels at standard protein load showed the increased expression of basic protein Pmg1p in the MeH protein-expressing cells (Figure 1, A-C, spot “?”); however, it appeared an artefact, because this result was not repeatable (see fold change error value for this spot in Table 3). Meanwhile in NEPHGE-based method, the corresponding protein spot was repeatable in all 2D gel replicas and did not show any considerable variation (Figure 1, D-F, spot “?” and Table 3). Instead of such artefacts, the NEPHGE-based method revealed repeatable and statistically significant increase of the expression of less abundant highly basic (~ pI 9) protein Sis1p in response to synthesis of MeH (spot “!” in Figures 1E, 2E and Table 3). Sis1p is type II HSP40 co-chaperone that interacts with the HSP70 protein Ssa1p, which is the most abundant cellular protein overexpressed during UPR-Cyto stress ([17] and Figure 1, spots a and b). It can be noted that Sis1p was also identified as overexpressed cellular protein during UPR-Cyto in another earlier study using misfolded YFP expression [20]. Therefore, the identification of Sis1p is a convincing result and expands our knowledge on UPR-Cyto stress.

Although we did not perform independent experiments at high protein load, the fold changes of the same overexpressed UPR-Cyto proteins were calculated from three technical replicates for comparison with standard load procedure (see Table 3, “High load” columns). Loading 2× protein amount on IPG strips had two effects on evaluation of differentially expressed UPR-Cyto proteins. Firstly, the standard deviations of fold changes of overexpressed Ssa, Sse1 and Hsc/Hsp82 proteins were highly increased. It shows that experimental gel-to-gel variation under these conditions highly exceeds biological variation. Such variation among technical replicates almost reaches the level of variation between different biological states (i.e. differential expression of UPR-Cyto proteins in MeH expressing versus control cells). High protein load on IPG strips also significantly lowered average fold change values for overexpressed Kar2p, Eno2p and partially degraded Ssa1/2p form (spot h). The latter was not recognized as the overexpressed protein due to overloading and poor resolution, which resulted in overlaped protein spots in corresponding 2D gel area (see Figure 2B, spot h). It is unclear why the well-shaped Kar2p spot showed the lower fold change, but it also seems incorrect result, because all other quantitative analyses including immunobloting (described below) showed considerably higher fold change values. Finally, abovementioned basic Gpm1 protein spot with false overexpression at standard protein load in IPG here showed an opposite result – repression. It once more confirmed that results in a basic zone of broad range IPG-based 2DE gels are unreliable. In summary, high protein load in pH 3–10 IPG strips was not suitable for the analysis of UPR-Cyto stress, therefore standard sample load is preferable for this method.

High protein load on NEPHGE gels resulted in lower fold changes and negligible standard deviations compared to 1× sample loading. Extreme fold change determined for Kar2p at standard protein load (9 ± 3.1) now was corrected to more reliable 2.5 ± 0.2 value, which better corresponds the Western blot result. This protein is not abundantly expressed under normal growth conditions; therefore, Kar2p spot is underrepresented in the control sample at lower protein amount, resulting in imprecise quantitative comparison. The only exception from lower fold changes of overexpressed UPR-Cyto proteins at high load analysis in NEPHGE-based method was the increase in fold change value of spot h (Figure 2E and Table 3). Possibly, in this case the result of 1x load experiment was also improved, because higher fold change of spot h is practically identical to earlier pH 4–7 IPG-based 2DE analysis. Taken together, high protein load in NEPHGE-based 2DE showed reliable results in the UPR-Cyto analysis with minimal experimental variation of differentially expressed protein spots. It seems worth to use the high protein load as optimal conditions for the routine analysis of yeast protein samples by NEPHGE-based 2DE technique, because it has only advantages over standard protein load.

### Verification of proteomic results by immunoblotting

**Figure 3:**
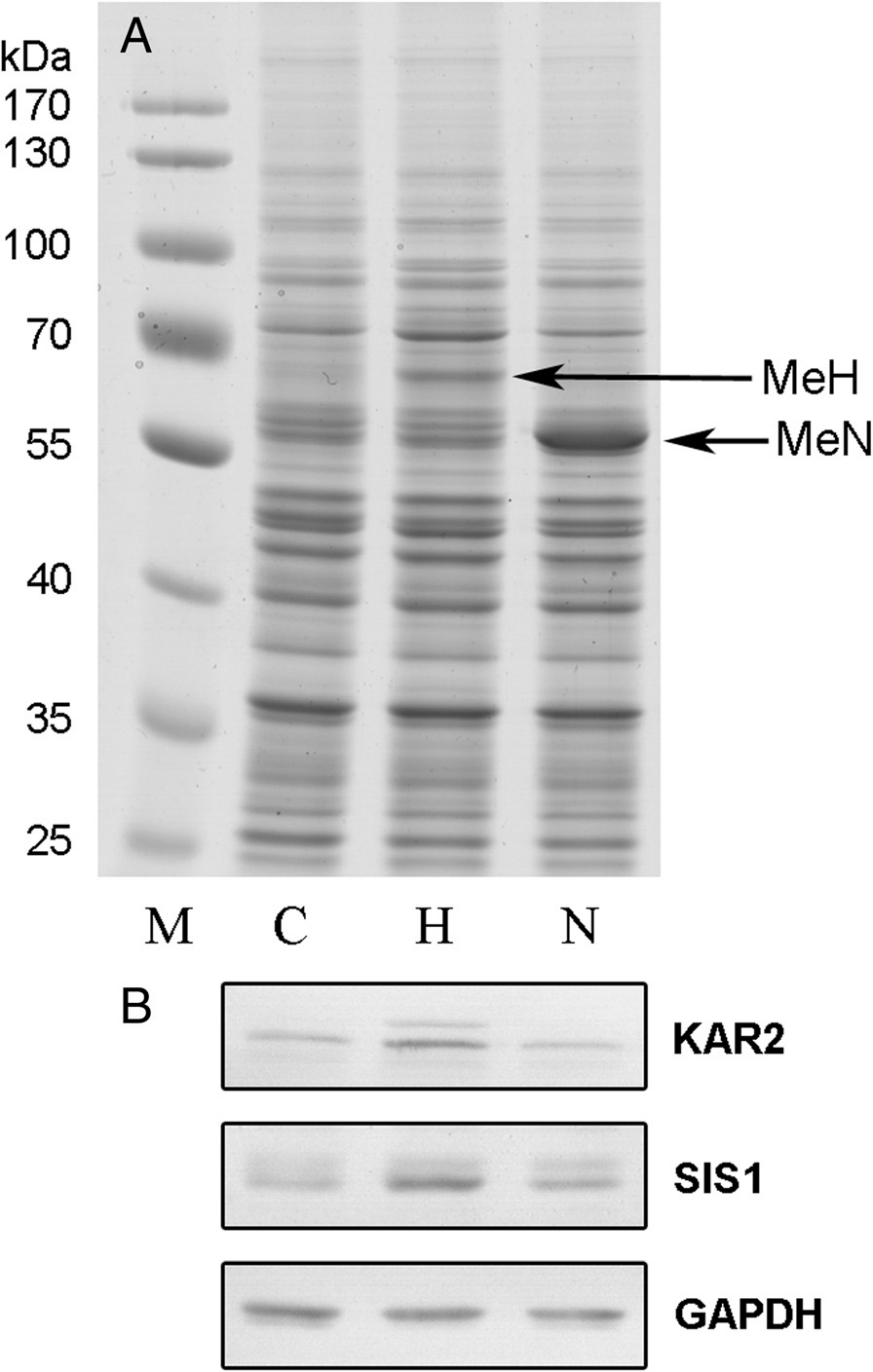
**Verification of proteomic results by immunoblot.** SDS-PAGE **(A)** and Western blot **(B)** analysis of crude yeast lysates are shown. Lysates were prepared from galactose-induced yeast cells of *S. cerevisiae* AH22 strain transformed with empty vector (control, lane C) or plasmids expressing MeH (lane H) or MeN (lane N). **(A)** Coomassie blue-stained gel. Solid arrows indicate bands of recombinant MeH (lane H) and MeN (lane N) proteins. Lane M - prestained protein ladder with molecular weights of bands indicated at the left. **(B)** Western blot analysis using the same samples transferred onto nitrocellulose membrane. The blots were probed with antibodies against yeast Kar2 and Sis1 proteins. GAPDH was used as loading control.

To confirm the results of 2DE study, we have done immunoblots using commercially available antibodies against two overexpressed UPR-Cyto proteins Kar2 and Sis1. Kar2p showed the highest overexpression in UPR-Cyto stress, but determined fold change greatly varied from 1.8 to 9 depending on 2DE technique and protocol (Table 3). Sis1p was the most important protein identified in this study, because it was a new protein involved in UPR-Cyto stress and exemplified the main advantage of NEPHGE over IPG in the analysis of highly basic proteins. Representative images of Western blot analysis of Kar2p and Sis1p expression in crude yeast lysates are shown in Figure 3. Alongside with the overexpressed main Kar2p form, immunoblot has also revealed an additional Kar2p band of slightly higher molecular weight in the cells expressing MeH protein, which induces UPR-Cyto (Figure 3B). Most likely it was a precursor of Kar2 protein with uncleaved signal sequence. Therefore, we have included both bands in calculation of Kar2p expression fold change. The results of three independent experiments showed 3.6 ± 0.5 increase of Kar2p expression in yeast cells expressing MeH protein. It corresponds to the fold change of Kar2p determined in our previous study using a narrow range pH 4–7 IPG-based 2DE method (Table 3). Western blotting using antibodies against Sis1p showed 1.9 ± 0.2 expression increase in MeH expressing yeast. It confirmed the overexpression of Sis1p during UPR-Cyto.

### General comparison of NEPHGE- and IPG-based 2DE methods

General characteristics of both methods are briefly summarized in Table 4. Considering all the data, NEPHGE-based method seems preferable in a broad range pH 3–10 gradient. The examples given above illustrate the essential differences between two methods in Tables 1 and 2: IPG 3–10 (Invitrogen) 2DE method is reliable only for the analysis of acidic proteins, whereas NEPHGE method produced acceptable results in entire pI range and was especially suitable for the analysis of basic proteins. By comparing only the results at high protein load, we should state that NEPHGE is by far better method, because all protein spot parameters are better than parameters obtained using IPG technique, even in the acidic range (see values in pI < 7 columns in Table 2). The results of differential expression proteomics experiment with UPR-Cyto stress confirmed that high sample load onto pH 3–10 IPG strips is unsuitable for studies of biological effetcs by 2DE. Therefore, it seems reasonable to discard from comparison high-load experiment with IPG strips and to directly compare IPG-based technique at standard 1x protein load with the NEPHGE-based method at 2x protein load. The comparison at optimal protein loading conditions (see Table 1 for IPG and Table 2 for NEPHGE) reveals very similar performance of both methods in acidic range with almost identical spot reproducibility and quality. Overall variation of spot volume parameters is also similar in this case, as higher variation averages in IPG-based 2DE are compensated by lower SD values. The only advantage of the NEPHGE method in this case is substantially larger protein amount resolved on 2D gel. As can be seen in Tables 1 and 2, it resulted in almost five fold higher number of separate acidic protein spots in NEPHGE (372 spots versus 79 spots detected by IPG). The parameters of detected protein spots (reproducibility, quality etc.) were nearly identical in both methods. It suggests that in the acidic zone both spot separation methods reached some optimal level, which results in similarly good parameters of resolved protein spots.

**Table 4:** General comparison of IPG- and NEPHGE- based 2DE methods

**Method**	**IPG**	**NEPHGE**
**Characteristics**		
Preparation of 1st dimension gels	Commercial gels; easy to prepare for IEF	Home-made gels; preparation requires skills
Procedure	Simple, easy to use	Complex, requires skills
Time for analysis	Fast, 2 days	Time-consuming, 5–6 days
Price	Cheap (Invitrogen)	Moderate (WITAvision)
Handling of 1st dimension gels	Handling of IPG strips is safe and easy	Gels are fragile, handling requires serious skills
Reproducibility	Well-reproducible in acidic, poor in a basic zone	Lower in acidic zone, but excellent in basic zone
Possible problems	Poor reproducibility of basic protein spots, protein capacity is limited	Handling difficulties, missing of some highly acidic protein spots
Protein capacity, effect of high protein load	Protein capacity is limited, quality of spots is worse at high protein load	Higher protein capacity, than in IPG gels; quality of spots is good at high load
General characteristic	Simple and easy to use method; ideal for 2-DE of acidic proteins. Drawback is poor reproducibility of basic protein spots.	Method requires skills; excellent for 2-DE of basic proteins. Analysis in acidic zone is satisfactory, but some spots are missed.

The intrinsic property of NEPHGE method is the higher protein capacity of 1st dimension gel than that of IPG gel of corresponding format. It is worth to discuss this in more detail, because it opens new opportunities. Detection of up to 500 good quality spots in a single small 2D gel by using Coomassie staining with relatively low sensitivity is a promising result. Taking into account experimental procedure and protein detection method, it seems difficult to achieve similar result using a broad range IPG strips. Thus, the main problem of pH 3–10 IPG strips seems to be limited amount of total protein that can be resolved into good quality spots on 2D gel. Moreover, significantly larger protein amount is lost during IPG-based 2DE procedure than in NEPHGE-based method even at standard protein load. In fact, there are several specific steps where the proteins are lost during IPG-based procedure. It was earlier shown that 20-55% of loaded protein is lost due to attachment of the proteins to reswelling tray during in-gel rehydration step [21]. Additionally, only 20%-51% of total protein amount loaded onto pH3-10 IPG strip was resolved onto 2D gel when complex protein mixture was analysed [21]. Cited study was performed using IPG strips produced by Amersham (now GE Healthcare). Therefore, it suggests that effects observed with Invitrogen strips in our study may be inherent to all pH 3–10 IPG strips in general. NEPHGE procedure does not include in-gel rehydration step (gels are casted and used fresh), and this could explain lower protein loss during 2DE. However, the most difficult thing is to explain why IPG strips are overloaded by much lower protein amounts than NEPHGE gels.

It should be noted that detection and analysis of large number of protein spots does not require the large amount of protein in the gel. Actually, we did not find any proteomic study on yeast proteins where a large number of protein spots (at least as high as in our study) was analysed by IPG-based 2DE using Coomassie staining method. For example, detection of ~1200 spots and creation of the 2D pattern as the yeast reference map was performed by using radioactive labelling [18]. Another 2DE study using radioactive labelling of yeast proteome reported detection of ~1100 protein spots [22]. Silver staining of large IPG-based 2D gels of yeast lysates resulted in visualisation of ~1000 spots per gel [19]. Yet another silver staining procedure was reported to visualise of ~1500 spots of yeast proteins; however, in that case a narrow range pH 4.7-5.9 IPG strip of the largest possible 25 cm length was used [23]. The use of silver staining for medium, 13 cm length, pH 3–10 IPG-based 2D gels resulted in detection and quantification of ~400 yeast protein spots per gel [24]. Finally, the most spots in yeast proteome were detected by using fluorescent SYPRO Ruby staining method, which resulted in >2,000 protein spots on each 2D gel [25]. However, the numbers of protein spots detected by IPG-based 2DE in any reported study seem small if compared to the potential of NEPHGE-based 2DE method. Over 10,000 protein spots were detected in one NEPHGE-based 2D gel using silver staining [12]. It is not clear why several times more sensitive protein detection methods are necessary if it is possible to detect the same thousand of proteins by simple Coomassie staining after loading much larger amount of protein mixture onto 1st dimension gel. This would be convenient for both quantitative analysis and mass spectrometry protein identification. Usually it is possible to unambigously identify any protein spot visualised by Coomassie staining. Most likely, 2DE analysis of a whole proteome using Coomassie stain was not being used due to limited protein capacity of IPG strips. In this context, NEPHGE technique may offer an improvement in 2DE-based proteomic studies.

When comparing narrow range pH 4–7 IPG (Invitrogen) and pH 3–10 NEPHGE systems, it is more difficult to conclude which method is better. Inability to detect some less abundant acidic proteins by the NEPHGE-based method at standard protein load was easily solved by increasing the amount of total cell protein. The essential drawback of the NEPHGE-based method in acidic protein analysis is disappearance of some differentially expressed protein spots. The examples here were Hsc82 and Hsp82 protein spots. However, NEPHGE-based method enabled identification of aforementioned basic differentially expressed Sis1 protein and this would compensate drawbacks in the acidic pI range. It should be mentioned that we have used anodic isoelectric focusing (AIF; sample applied to the anodic side of the gel) according to the NEPHGE technique developed by Klose [3], in contrast to cathodic isoelectric focusing (CIF; sample applied to the cathodic side of the gel) developed by O’Farrell [4]. It was reported earlier that when using CIF, a whole class of proteins (very basic proteins) is lost, whereas when using AIF, a certain amount of each protein in a protein class (very acidic proteins) do not enter the gel [11,12]. Our results suggest that the broad range pH 3–10 IPG-based 2DE method suffers from the same limitations (loss of the very basic proteins) as CIF technique of the NEPHGE method. Here is important to note that these specific problems are rather small if compared to the main drawback of a basic 2DE method itself. A lot of proteins do not enter any 2D gels at all. Usually a very few membrane proteins are detected by 2DE. Moreover, there are also other protein classes that are not presented on 2D gels. Good example is recombinant viral proteins MeH and MeN with the pIs of ~6.6 and ~5.2, respectively. In this study they were overexpressed in the yeast cells and are presented as strong bands in SDS-PAGE of crude yeast lysates (Figure 3A). If all proteins from whole cell lysates would enter 2D gels, MeH and MeN should be presented at microgram amounts. However, no traces of these proteins were observed in 2D gels using both 2DE methods (Figures 1 and 2). It is evident that the loss of more than a half protein amount during 2DE procedure [21] is rather specific and a lot of proteins are totally lost from the samples. Taken together, there is no ideal technique for 2DE method, because all techniques have some drawbacks. In our case, it seems the most efficient way to be the usage of large format NEPHGE gels for a broad range pH 3–10 analysis, whereas in the acidic range the analysis could be doubled by the narrow range IPG mini-gels (pH 4–7 or pH 4.5-5.5 etc.).

## Conclusions

First dimension IPG (Invitrogen) and NEPHGE (WITAvision) techniques were directly compared in two-dimensional gel electrophoresis experiment using the same format mini-gels and the same samples of yeast whole cell lysates. Comparison of a broad range pH 3–10 gradient based 2DE methods suggests that NEPHGE-based method is preferable. IPG 3–10 (Invitrogen) 2DE method is reliable only in analysis of acidic proteins, because in basic side of 2D gels the results are not reproducible; meanwhile, NEPHGE method is suitable in the entire pI range and especially efficient for the analysis of basic proteins. In this study this was exemplified by identification of highly basic protein Sis1p overexpressed during UPR-Cyto stress in yeast cells. This protein was convincingly identified as differentially expressed protein by using NEPHGE, but not IPG based 2DE method. Overexpression of Sis1p was confirmed by immunoblot analysis. Nevertheless, the narrow range pH 4–7 IPG (Invitrogen) technique is a better method for the analysis of acidic proteins. Considering all the results derived from tested techniques, it seems the most efficient way is to use large format NEPHGE gels for a broad range pH 3–10 analysis, whereas in acidic range the analysis could be doubled by the narrow range IPG mini-gels (Invitrogen).

## Methods

### Plasmids, yeast strain, media and growth

Three plasmids were used in this study: pFGG3-MeH (for inducible expression of MeH protein causing UPR-Cyto stress in yeast), pFGG3 (empty control vector) and pFGG3-MeN (additional control for inducible expression of MeN protein, which does not cause the stress response in yeast). Generation of these DNA constructs were described previously (see [17] for pFGG3-MeH and [26] for pFGG3 and pFGG3-MeN).

The plasmids were used for the transformation of the *S. cerevisiae* strain AH22 (*MATa leu2-3 leu2-112 his4-519 can1* [*KIL-o*]) as described previously [27]. Yeast culture media, growth and induction of *S.cerevisiae* transformants were exactly the same as reported in earlier study [17]. After induction of viral protein expression, transformed cells were harvested by centrifugation and stored at -70°C.

### Design of the study

The aim of this study was to directly compare the first dimension IPG and NEPHGE techniques in two-dimensional gel electrophoresis (2DE) method and evaluate their impact on the results of biological experiment. Commercially available systems “ZOOM IPGRunner” from Invitrogen and a “WitaVision g1D” from WITA GmbH (Teltow, Germany) were chosen for IPG and NEPHGE, respectively. The same platform including pI range (pH 3–10 gradient) and the gel length (7 cm mini-gels) was used for both methods.

The study was designed according to the main tasks: (i) to evaluate experimental variation in both 2DE techniques by running the same samples several times; (ii) to assess biological variation in protein expression during UPR-Cyto stress in yeast cells using both 2DE methods and thereby compare their efficiency in the concrete biological experiment. The biological material was essentially the same as reported previously [17] except that here we have used only measles virus proteins for the expression in yeast (i.e. mumps virus proteins were not used). Briefly, the UPR-Cyto stress was induced by the expression of MeH protein and the pattern of cellular proteins resolved by 2DE was compared to protein pattern from the control cells transformed with empty expression vector pFGG3. In addition, the yeast cells expressing MeN protein, which does not induce cellular stress, were used as internal control in this study. Experimental variation in both 2DE methods was evaluated using the same samples from three experimental variants (expressing MeH, MeN or control cells). This analysis was doubled by loading 1x and 2x amounts of protein samples (standard and high load conditions, respectively). 2DE of the same samples (from one independent experiment) was repeated three times and various parameters were calculated for both methods as is shown in Tables 1 and 2. Biological variation in cellular protein expression was assessed by performing independent experiments (transformation of yeast cells with vectors, growing yeast cells, induction of viral protein expression, preparation of whole cell lysates and 2DE with subsequent gel staining and image analysis) at standard 1x protein load. Fold changes for differentially expressed proteins were calculated from at least three independent experiments at standard conditions and the results are given in Table 3. In addition, fold changes of the same protein spots were calculated from three replicas of one independent experiment at high protein load and the values were also included in Table 3 for comparison.

In principle, all operations in both 2DE methods were performed in parallel except for 1st dimension electrophoresis (IEF – isoelectric focusing). The same samples were applied onto IPG strips and NEPHGE gels. After IEF in different system equipments, the 2nd dimension electrophoresis (SDS-PAGE) was performed in parallel for corresponding IPG and NEPHGE samples (i.e. the SDS-PAA gels were casted and run simultaneously). All 2D gel staining and image analysis procedures were also identical for comparable IPG and NEPHGE-based 2DE samples. Therefore, the results should be influenced only by differences in the 1st dimension techniques and this enables their direct comparison.

### Preparation of yeast lysates for 2DE

10**–**20 mg of cell pellets were collected into a 1.5 ml microcentrifuge tube by centrifugation, washed with distilled water and stored frozen at -70°C. After storage cells were quickly thawed and resuspended in 10 volumes (vol/wt) of denaturing IEF buffer containing 7 M urea, 2 M thiourea, 2% CHAPS detergent, 2% ampholytes (pH 3–10, GE Healthcare), 0.002% Bromphenol Blue and 75 mM DTT (added just before use). Note, that IEF buffer composition (given above) was suitable for both methods according to manufacturer’s (Invitrogen and WITA, respectively) recommendations. An equal volume of glass beads was added and the cells were lysed by vortexing at high speed, 8 times for 30 sec, with cooling on ice for 10 sec followed by keeping 30 sec at room temperature between each vortexing. Then cell debris was removed by centrifugation at 16000 × g for 15 min. at 16°C. Supernatants (whole cell lysates) were applied onto 7 cm length IPG strips or onto 7 cm NEPHGE 1st dimension gels. Protein concentrations were determined by Roti-Nanoquant Protein-assay (Carl Roth Gmbh.), which is a modification of Bradford’s protein assay. Additionally, protein concentrations in the supernatants were checked by SDS-PAGE followed by staining with Coomassie Brilliant Blue R-250 and evaluation of total protein amount in 1D gel lanes using the ImageQuant TL 1D gel analysis software (GE Healthcare). Samples were diluted with IEF buffer if necessary and equal protein concentrations were used for two-dimensional gel electrophoresis.

For high-load 2DE experiments, more concentrated samples were prepared by using less volume of denaturing IEF buffer. Cells were resuspended in 5 volumes (vol/wt) of denaturing IEF buffer and further preparation procedure was the same as described above.

### Running the first dimension

For comparative analysis, the same samples from expressing MeH, MeN or control cells were run by both IPG and NEPHGE methods. The first-dimensional separation of proteins was performed according to manufacturer’s (Invitrogen and WITA, respectively) recommendations. Briefly, IPG strips (ZOOM strips pH 3-10NL, Invitrogen) were used for IEF in Invitrogen ZOOM IPGRunner System. 50 or 100 μg of the protein from whole cell lysate was diluted to 155 μl by IEF buffer and applied onto IPG strip following rehydration overnight. Next day the ZOOM IPGRunner Mini-Cell was assembled and IEF was performed using “PowerEase 500 Power Supply” (Invitrogen) with the following running conditions: 200 V for 20 min; 350 V for 10 min; 500 V for 4 hrs. Finally, a higher voltage step at 2000 V was performed as recommended by manufacturer (for 2 hrs, the power supply “Consort EV233”). Focused IPG strips were stored in a sealed container at -70°C. Before 2nd dimension, the strips were incubated in equilibration buffer (50 mM Tris–HCl pH 8.8, 2% SDS, 6 M urea, 30% glycerol, 0.002% Bromphenol Blue) containing, in course, reducing (75 mM DTT) and alkylating (125 mM 2-iodoacetamyde) agents (treated for 15 min. by both). Equilibrated strips were applied onto SDS-polyacrylamide gels and SDS-PAGE was run for the second dimension.

NEPHGE was made with a non-linear pH3-10 gradient formed by carrier ampholytes. The mixture of carrier ampholytes and IEF gel solution composition was made according to Klose and Kobalz, 1995 [12]. Briefly, ampholytes of pH5-6.5 were at the highest concentration followed by ampholytes of pH4-5 and pH6.5-8, then with further expansion of pH gradient. Accordingly, it gives wider separation zone at the pH5-6.5, followed by pH4-5 and pH6.5-8. It is similar to used pH3-10NL IPG strips; however, small shift may be observed.

First dimension NEPHGE was performed according to the protocol of the manufacturer, using a set of standardized materials (from WITA Gmbh). Briefly, two gel solutions were cast in succession in a vertical device for preparation of the two-layered rod gels of the first dimension (quantities sufficient for a total of eight rod gels): 1.5 ml of separation gel solution plus 36 μl of 0.8% ammonium persulfate (APS) was prepared for polymerization of the first gel layer, and 600 μl of cap gel solution (WitaVision) was mixed with 15 μl of 0.8% APS for formation of the second gel layer of the rod gels (all solutions were degassed by sonication). For complete polymerization, the gels of the first dimension were held at room temperature for 30 min and then kept in a damp chamber for additional 72 hr. The first-dimensional separation of proteins in the rod gels was performed in a vertical electrophoresis device according to the operating instructions of the manufacturer (WitaVision). Briefly, the lower chamber of the device was filled with 400 ml of degassed cathode buffer (prepared on a 40°C heating plate, containing 20 g of glycine, 216 g of urea, 200 ml of aqua dest, filled up to 380 ml; and the addition of 20 ml of ethylenediamine). Following fixation of the rod gels in the device, the sample solutions containing 30 or 100 μg of the protein from whole cell lysate in agarose-supplemented ampholyte phosphate buffer were applied to the anodic sides of the capillary gels, and the remaining volumes of the capillary glass tubes were then covered with a sample stabilizing overlay solution (WitaVision). Subsequently, 400 ml of degassed anode buffer were applied (solution of 72 g of urea, 250 ml of aqua dest, filled up to 380 ml; addition of 20 ml of 85% phosphoric acid) to the upper chamber of the device, and the electrophoretic separation of the first dimension was started by using the following sequence of programmed running conditions: 100 V for 1 hr 15 min; 200 V for 1 hr 15 min; 400 V for 1 hr 15 min; 600 V for 1 hr 15 min; 800 V for 10 min; 1000 V for 5 min. After the termination of electrophoresis, the rod gels were carefully pushed out of the glass tubes onto plastic rails, and adaptation to the conditions of the second dimension was achieved by a series of three 15-min equilibrations in a corresponding equilibration buffer containing 75 mM DTT, followed by equilibration in the same buffer with 125 mM 2-iodoacetamyde. The equilibrated rod gels of the first dimension were stored at -70°C before application to the second dimension of the 2DE system.

### Running the 2nd dimension (SDS-PAGE), fixing and staining of 2D gels

For separation in the second dimension of 2DE, standard SDS-PAGE was performed with 11% (w/v) polyacrylamide gels using a Minigel-Twin units (Biometra). Briefly, the IPG strips and rod gels of the first dimension were gently transferred from equilibration and storage rails to the top of the stacking gel zones and covered with 0.5% (w/v) agarose to fix the rod gels. The electrophoresis running conditions of the second dimension separation were set as follows: 15 mA per gel (~100 V) for ~ 15 min (untill the dye reached resolving gel); 30 mA per gel (voltage gradually rises up to 200 V limit) for about 1 hr, until the bromophenol blue front reached the bottom of the gel.

After 2DE protein separation was complete, gels were fixed in Fixation Solution (50% ethanol, 40% HPLC grade water, 10% acetic acid) for at least 1 hr under gentle agitation at room temperature (RT) and stained with Coomassie Brilliant Blue R-250 (50% ethanol, 10% acetic acid, 0.1% Coomassie BB R-250, 40% HPLC grade water) over night under gentle agitation at RT. Next day the gels were destained in Destaining Solution (5% ethanol, 12.5% acetic acid in HPLC grade water) for 1 hr under gentle agitation at RT followed by further destaining with Storage Solution (7% acetic acid in HPLC grade water) for 4 hr (at least 2x exchange of solution) at RT. Then the 2D gels were scanned with ImageScanner III (GE Healthcare) and stored sealed in plastic pouches at 4°C.

### Analysis of 2D gel images

All 2D gels in this experiment were scanned with calibrated ImageScanner III (GE Healthcare) under the same settings: blank filter, transparent mode and 300 dpi resolution. The gels that were run in parallel have been scanned simultaneously (usually six gels at once – three from IPG-based and three from NEPHGE-based 2DE; original scanned image of one of the replicas is shown in Figure 2). Then the image was resolved into separate 2D gel images and these were imported into 2D gel analysis software. 2D gel images were analysed using the ImageMaster 2D Platinum 7.0 software (GE Healthcare). Protein spots were detected automatically by setting the same parameters (smooth, saliency and min area) for all analysed 2D gels. Artefact spots (mostly near the boundaries of the gels) were deleted manually in every 2D gel with detected spots. Then gels were matched in separate small groups of three gels (e.g. IPG 3–10 analysis of Control, MeH and MeN variants) followed by matches between the groups according to required comparison. 2D gel images or match sets were grouped into classes according to the task of analysis (e.g. the analysis of experimental variation between replicas of the same samples). Various comparisons and calculations of parameters were performed as indicated in the legends of Tables 1, 2 and 3. All 2D gels were divided into acidic and basic parts according to the position of known cellular proteins with near neutral pIs. The line of neutral pI 7.0 was applied to all gels at the same position of protein 2D pattern as it is shown in Figures 1 and 2. Then acidic or basic gel parts were selected and required calculations for acidic and basic protein spots performed as it is indicated in Tables 1 and 2. Differentially expressed cellular proteins during UPR-Cyto stress were evaluated by calculating “fold change” - the ratio of %Vol between spots of MeH expressing and control cells, respectively. Fold changes given in Table 3 represent data from three independent experiments (values are averages ± SD). Differentially expressed spots were also analysed in internal control samples from cells expressing MeN protein. The expression level of differentially expressed protein spots indicated by arrows in Figures 1 and 2 was similar in both control and MeN expressing cells (data not shown).

### Protein identification

The protein identification was carried out at the Proteomics Center in the Institute of Biochemistry (Vilnius, Lithuania) by means of tryptic digestion and mass fingerprinting. Tryptic digestion was performed according to earlier described procedure [28]. Briefly, protein spots were excised from the gel and cut into 1×1 mm pieces. Gel pieces were destained with 200 μl of 25 mM ammonium bicarbonate in 50% acetonitrile (ACN), dehydrated with ACN and incubated with 40 μl 10 ng/μl of trypsin solution in 25 mM ammonium bicarbonate over night at 37°C. Next day, peptides were extracted with 2 × 100 μl 5% trifluoroacetic acid (TFA), lyophilized and dissolved in 3 μl 0.1% TFA in 50% ACN. Samples were applied to 384-well MALDI plate. 0.5 μl of sample were overlayed with 0.5 μl of matrix (alpha-cyano-4-hydroxycinnamic acid, 4 mg/ml 50% ACN with 0.1% TFA).

Proteins were identified by matrix-assisted laser desorption/ionization (MALDI) mass spectrometry using 4800 MALDI TOF/TOF mass spectrometer (AB/Sciex). Peptide mass spectra were acquired in reflector positive ion mode in m/z range 800–4000 Da, 400 laser shots were summed for each sample with mass accuracy ±50 ppm. MS/MS spectra for dominating peptides were acquired in positive mode, ion collision energy was set to 1 keV, 500 laser shots were accumulated for each spectrum with mass accuracy ±0.1 Da. Proteins were identified in the TrEMBL database (3-23-10 release) using the Mascot algorithm. Summary of protein identification data is provided in Additional file 1.

### SDS-PAGE and Western blotting of crude yeast lysates

Proteomic results were verified by immunobloting of identified overexpressed Kar2 and Sis1 proteins. Crude yeast lysates were prepared for SDS-PAGE as described in [17]. One gel copy was stained with Coomassie brilliant blue R-250, and another was blotted onto the nitrocellulose membrane Hybond™ ECL (Amersham, UK) as described in [29] and incubated with antibodies according to the manufacturers’ recommendations. The primary antibodies used were rabbit anti-Kar2 (y-115, sc-33630, Santa Cruz Biotechnology), rabbit anti-Sis1 (COP-COP-080051, Cosmo Bio Co, Japan) and mouse anti-GAPDH Loading Control Antibody (GA1R, Thermo Scientific). Horseradish peroxidase (HRP)-labelled goat anti-rabbit and goat anti-mouse IgG conjugates (Bio-Rad, 172–1019 and 172–1011, respectively) were used for the detection of specific antibody-binding. GAPDH was used as loading control.

Quantitative evaluation of protein bands in immunoblots was performed using the ImageQuant TL 1D gel analysis software (GE Healthcare). Intensity values of Kar2p and Sis1p bands were normalized to their respective GAPDH bands. Expression fold changes were calculated from three independent experiments as averages ± standard deviation (SD).

## Competing interests

The authors declare that they have no competing interests.

## Authors’ contributions

RS was involved in all aspects of the experimental design, data collection, analysis and interpretation, and drafted the manuscript. RR carried out the analysis of 2D gel images. RZ participated in sample preparation, 1st dimension NEPHGE, 2nd dimension electrophoresis experiments and made immunoblots. EČ carried out the 1st dimension IPG-based IEF and participated in 2nd dimension electrophoresis experiments. All authors read and approved the final manuscript.

## Supplementary Material

Additional file 1 **Protein identification data.** Identification data of protein spots “h”, “?” and “!”. Identified proteins are highlighted by yellow colour. Click here for file
